# Phase Transition of Gels—A Review of Toyoich Tanaka’s Research

**DOI:** 10.3390/gels8090550

**Published:** 2022-08-30

**Authors:** Masayuki Tokita

**Affiliations:** Department of Physics, Faculty of Science, Kyushu University, 744 Motooka, Fukuoka 819-0395, Japan; bm10kita_mini@icloud.com

**Keywords:** volume phase transition of gel, polymer-solvent interaction, equilibrium behaviors, dynamics of volume phase transition of gel

## Abstract

In 70’s, the extensive studies about the gel science has begun with the discovery of the volume phase transition of gel at the physics department of Massachusetts Institute of Technology. After the discovery of the volume phase transition of gel, the phenomenon was extensively studied and advanced by the discoverer, the late Professor Toyoichi Tanaka, who deceased on 20 May 2000 in the halfway of his research. In this paper, we would like to review his research to clarify his deep insight into the science of gels.

## 1. Introduction

Gel is one of polymer system that consists of the three-dimensional cross-linked network of polymers and a huge amount of solvent. It has been well established that the polymer gels swell and/or shrink in response to the changes of environmental conditions that the gel is surrounded. The equilibrium swelling behaviors of the hydrogel under various external conditions have been studied extensively and the results are summarized in Flory’s “Bible” [1]. It is shown by Flory that the equilibrium swelling ratio of the gel is determined by the combination of several interaction parameters that contribute to the osmotic pressure of the gel; the rubber elasticity, the interaction between polymer and solvent, and the degree of ionization of the polymer network of gel. It is found that the osmotic pressure of the gel that calculated theoretically well explains the experimental results of the swelling behavior of ionized gels that consist of methacrylic acid and divinylbenzene [2,3]. Besides, it is also reported that the polyelectrolyte gel shows a discontinuous transition behavior in its length-force curve under constrained conditions [4]. Therefore, the theoretical framework of the swelling behavior of gel are already constructed in early 50’s with many experimental results. The concept of the volume phase transition of the polymer gel are suggested theoretically about twenty years after Flory [5]. However, it took further ten years for the observation of the volume phase transition in actual system of polymer gels by the late Professor Toyoichi Tanaka of Massachusetts Institute of Technology. Professor Tanaka was an experimental scientist but he was also passionate about the theoretical study. As seen in the later, his research papers are the beautiful collaboration of the experimental study and the theoretical analysis of the experimental results. This feeling of excitement is always noticeable in his professional talks, and it is clearly seen in many of his research papers.

In the end of the 70’s, he discovered the volume phase transition phenomenon of polymer gel. This discovery became one of the most frequently cited works of Tanaka in the period. After the discovery, numerous followers all over the world studied various phase transitions in gels. It was, however, Tanaka whose comprehensive vision made the difference. He examined phase transitions in many different types of gels systematically. The results are summarized in four types of weak interactions that operate in water. He has achieved a systematic understanding, namely, when gel shows the discontinuous volume change it is a first order phase transition. On the other hand, if the volume change of the gel is continuous, it is a second order transition. This gave rise to industrial applications of responsive gels, such as the heat sensitive gel, the gel that sense the electric and magnetic fields, the light sensitive gel, responsive gels to pH and chemicals. In this review, we would like to looking back the discovery of the phenomenon and the advancements of the phase transition of gel through Tanaka’s scientific research. We believe that such a review is still worth publishing for the young scientists who wish to start studying the gel sciences.

## 2. Equilibrium Property of Gel

### 2.1. Discovery of the Volume Phase Transition of Gel

When a gel is soaked into a solvent, the gel attains a thermodynamic equilibrium state. We recognize that the gel is in the equilibrium state when the gel reaches at an equilibrium volume. The volume of the gel, namely, the equilibrium state of the gel is uniquely determined by a set of environmental conditions. We, thus, define the volume ratio of gel, $V/V_{0}$, as the measure of the volume of the gel at an equilibrium state where *V* and $V_{0}$ are the volume of the gel at an equilibrium state and that at the reference state. The choice of the reference state is rather arbitrary. Hereafter, we choose the diameter, $d_{0}$, of the cylindrical capillary as the reference state, which we soaked into the monomer solution and then the gelation reaction takes place. The diameter *d* of the rod shaped gel thus obtained are measured under various experimental conditions. Then the volume swelling ratio of the gel is calculated by ${\left(d/d_{0}\right)}^{3}=V/V_{0}$. The ratio of the diameters of the gel $d/d_{0}$ is occasionally employed as the swelling ratio of the gel. It should be noted here that the reference state itself is a function of experimental conditions such as the composition of monomer and cross-linker, the gelation temperature, and so forth. The swelling behaviors of gels are measured and discussed thus far. The studies that have been made before the discovery of the volume phase transition of gel are described in the Flory’s book [1].

**Figure 1:**
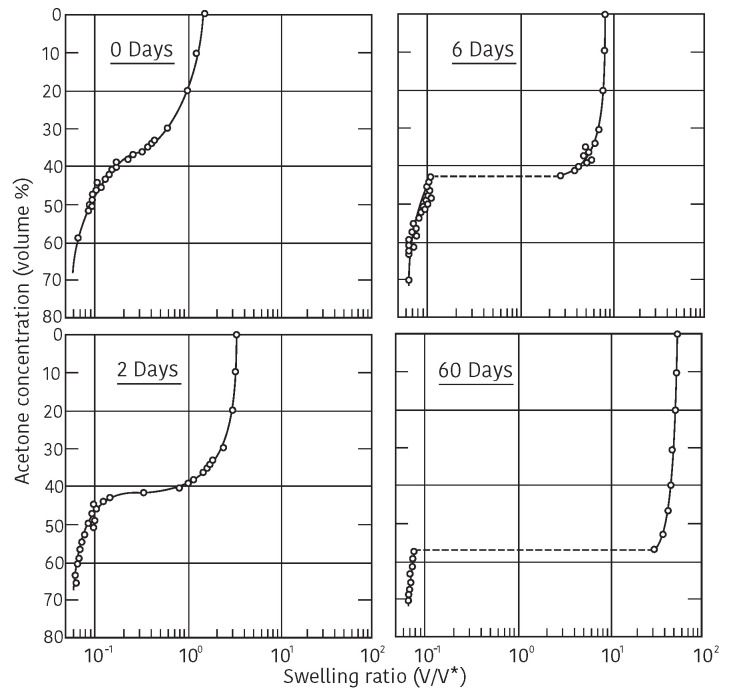
The volume phase transition of ionic poly(acrylamide) gels. The equilibrium swelling ratio of the gels in the mixed solvent system of water and acetone. The time of hydrolyzed reaction are given in the figure as, for instance, 6 Days. Reproduced with permission from Ref. [8]. © 1986, The Physical Society of Japan.

In 1978, however, Tanaka discovered a new phenomenon that the volume of poly-(acrylamide) gel changes discontinuously at a certain conditions [6]. In this study, he realized the important roles played by the ionic group on the polymer chain. He, then, studied the swelling behaviors of gel by changing the ionization of the polymer network of the gel systematically [7]. The results are shown in Figure 1. The hydrolyzed poly(acrylamide) gels in alkaline solution are employed in this study. The degree of ionization of polymer chain is changed by changing the duration of hydrolyze reaction. The results obtained in these earlier studies are, therefore, not quantitative enough. The quantitative studies on the effects of the ionic group in the polymer chain is made later. However, the characteristic features of the volume phase transition of gel are well shown in these experimental results: for instance, the presence of the critical point, the temperature dependence of the transition point, the degree of volume change at the transition point, and so forth. The behaviors of the characteristic features can be intuitively explained by the effects of the ionic group in the polymer network. This is the discovery of the volume phase transition phenomenon in polymer gels. He, then, establish the theory of the volume phase transition of gel to describe the experimental results.

### 2.2. How the Volume Phase Transition of the Gel Occurs

#### 2.2.1. Analogy with the Gas-Liquid Phase Transition

It may be worth glancing over the similarity between the gas-liquid phase transition and the volume phase transition of gel. The phase behavior of the gas-liquid system, for instance water, is well understood and the phase diagram in (V−p) space has been established. It is well known that water is in the liquid state below 100 °C under the atmospheric pressure. It, however, becomes vapor above 100 °C at a pressure of 1 atm. The volume of water reversibly changes about 1700 hold when water transforms into vapor and *vice versa*. The spatial distribution of the density of water fluctuates in time and space. The fluctuations of the density diverges at the phase transition point. Microscopically, the transition point is determined by the balance of the thermal motion and the attractive interaction, which is called as the van der Waals interaction for the sake of simplicity. The thermal motion of the water molecules becomes dominant at higher temperatures, say above 100 °C, that promote the change of water molecule from the liquid state to the vapor state. On the other hand, the attractive force by the van der Waals interaction becomes dominant at lower temperatures, and hence, the liquid state of water is preferable. This is a rough sketch of the gas-liquid phase transition.

Tanaka thought that similar phenomenon occur in polymer gels if the segment of polymer chain in the polymer network of gel is regarded as the “gas” molecule. The appearance of the phase transition in the gel is, however, considerably modified from that of the gas-liquid phase transition because the “gas” molecule of the polymer gel are connected each other through the chemical bonds including the cross-links to make an infinite polymer network of the gel. The segments of polymer network are, therefore, forbidden to expand infinitely and hence the equilibrium volume is limited. Besides, the elastic property of the gel also deforms the appearance of the phase transition since the elastic property is a characteristic feature of solid and both the gas and liquid does not show the elasticity except for the bulk modulus. For instance, the pattern that appears on the surface of the swelling gel is a typical example of the effects of the elasticity of the polymer network. The patterns that appear in the shrinking process are also induced by the complicated interactions between the elasticity, the destruction of the polymer network, and the over cooling effects of the system. These points are shortly discussed later. However, we get a benefit from the presence of the polymer network since we can study the phase transition of the gel only by the naked eye observation of the gel; a so-called “five cents experiment”.The fact that the volume phase transition of gel occurs in liquid solvent under atmospheric conditions is another advantage of the studying the volume phase transition of gels. In addition to these benefits of the polymer gel, the elastic properties of the gel due to the polymer network gives rise the unique behaviors to the phase transition of the gel such as the formation of patterns in both the swelling and the shrinking processes.

#### 2.2.2. Analogy with the Gas-Liquid Phase Transition

Tanaka established the equation of state of the gel taking into account following four forces that create the osmotic pressure of the gel [1,7].

1. Osmotic pressure due to the rubber elasticity; $\pi_{\mathrm{re}}$.
2. Osmotic pressure due to the interaction between polymer and solvent; $\pi_{\mathrm{ps}}$.
3. Osmotic pressure of counter ions; $\pi_{\mathrm{ion}}$.
4. Osmotic pressure due to the mixing entropy; $\pi_{\mathrm{mix}}$.

According to the similarity of the configuration between the gel and the rubber, the osmotic pressure, π, due to the rubber elasticity is calculated on the basis of the Gaussian statistics.

(1) $$\pi_{\mathrm{re}}=\nu kT\left[\left(\frac{V_{0}}{2V}\right)-{\left(\frac{V_{0}}{V}\right)}^{1/3}\right]$$

Here, ν represents the number of elastically active chain in the unit volume of the gel at the reference state and $V/V_{0}$ is the swelling ratio of gel, respectively.

The volume of gel is also changed by the interactions between the segments of the polymer and the solvent molecules. When the affinity between polymer segments is preferable than that between the polymer segment and the solvent molecule, the gel tends to shrink. In contrast, the gel tends to swell if the affinity between the polymer segment and the solvent molecule is preferable than that between polymer segments. The osmotic pressure due to the interactions between polymer segments and solvent molecules are reasonably expressed by Flory-Huggins theory of solutions as

(2) $$\pi_{\mathrm{ps}}=-\left(\frac{\Delta F}{2\upsilon_{0}}\right)\phi^{2},$$

where ΔF is given by

(3) $$\Delta F=\Delta F_{\mathrm{PP}}+\Delta F_{\mathrm{SS}}-2\Delta F_{\mathrm{PS}}.$$

Here, ϕ and $\upsilon_{0}$ represent the volume fraction of the polymer network in the gel and the volume of solvent molecule. The free energies $\Delta F_{\mathrm{PP}}$, $\Delta F_{\mathrm{SS}}$, and $\Delta F_{\mathrm{PS}}$ are the free energy of contacts between two polymer segments, between two solvent molecules, and between polymer segment and solvent, respectively. The volume fraction of the polymer network of gel is related with the volume of the gel as $\phi =\phi_{0}\left(V_{0}/V\right)$ where $\phi_{0}$ is the volume fraction of the polymer network at the reference state. The factor $\phi^{2}$ represents the probability of contact between two polymer segments and ΔF changes with the composition of the solvent. Here, Tanaka focus his attention to two body interaction.

The osmotic pressure due to the ionizable group in the polymer chains of the polymer network of gel plays crucial roles in the discontinuous volume change of the gel. When the gel, in which the polymer chain of the gel contains ionic groups, is soaked in pure water, the ionic groups tend to dissociate into positively and negatively charged groups. The dissociation of ionizable groups is governed by the dissociation equilibrium. Both the positive charge and the negative charge emerges in the gel but the numbers of positive charge, $n_{+}$, and negative charge, $n_{-}$, are the same by the conservation law of charges. Accordingly, the number of excess charge in the gel, $n_{\mathrm{gel}}$, is always zero, $n_{\mathrm{gel}}=\left(+e\right)n_{+}+\left(-e\right)n_{-}=0$ where *e* is the elementary electric charge. The gel is, therefore, always electrically neutral as a whole even it contains the ionic group in the polymer network of gel. In the earlier studies, the poly(acrylamide) gel was hydrolyzed to introduce the ionic group in the gel by which acrylamide is transformed into acrylic acid. A part of the polymer chain of the hydrolyzed poly(acrylamide) gel is negatively charged. The positive counter ion of the proton emerges in the gel as a result of the dissociation. The protons, thus, freely diffuse within the polymer network of the gel. The counter ion, however, may not diffuses out of the gel by the conservation law of charges. The counter ions, thus, confined within the polymer network of gel create a pressure to the *wall* of the gel. The osmotic pressure due to the confined counter ion in the gel is given as follows.

(4) $$\pi_{\mathrm{ion}}=f\nu kT\left(\frac{V_{0}}{V}\right)$$

Here, *f* represents the number of counter ion that emerges from an elastically active polymer chain of the polymer network. and hence, fν corresponds to the number of counter ion in a unit volume of the gel in the reference state.

The thermodynamic relationships for the polymer solutions are discussed by Flory using the lattice model. According to Flory, the entropy of mixing, ΔS, is approximately given as follows [1].

(5) ΔS=nln(1−ϕ)

Here, *n* is the number of the solvent molecule. Then, the osmotic pressure due to the entropy of mixing is obtained.

$$\pi_{\mathrm{mix}}=-\frac{\partial kT\Delta S}{\partial V}$$

The equation above can be rewritten as follows using a relationship $n=\left(V/\upsilon_{0}\right)\left(1-\phi \right)$.

(6) $$\pi_{\mathrm{mix}}=\frac{kT}{\upsilon_{0}}\left[\ln\left(1-\phi \right)+\phi \right]$$

We, thus, obtained all relationship that contribute to the osmotic pressure of gel. The equation of state of the gel is, then, obtained by summing up the Equations (1), (2), (4) and (6).

(7) $$\frac{\pi }{kT}=\nu \left[\left(f+1/2\right)\left(\frac{V_{0}}{V}\right)+{\left(\frac{V_{0}}{V}\right)}^{1/3}\right]-\frac{\ln(1-\phi )+\phi }{\upsilon_{0}}-\left(\frac{\Delta F}{2\upsilon_{0}kT}\right)\phi^{2}$$

The total osmotic pressure, $\pi =\pi_{\mathrm{re}}+\pi_{\mathrm{ps}}+\pi_{\mathrm{ion}}+\pi_{\mathrm{mix}}$, is the sum of each contribution. The properties of gel at an equilibrium state can be described by the Equation (7). The Equation (7) is constructed on the assumption of homogeneous gel. This limits the application of the theory because the volume phase transition proceeds through biphasic heterogeneous states although the initial and the final states of the gel are homogeneous. The patterns that appear in the swelling and the shrinking processes of the gel are typical examples.

#### 2.2.3. Theoretical Swelling Curve of Gel

The swelling curve of the gel can be deduced from Equation (7) by setting that the osmotic pressure is zero, π=0, at an equilibrium state of gel.

(8) $$1-\frac{\Delta F}{kT}=\frac{2\nu \upsilon_{0}}{\phi^{2}}\left[{\left(\frac{V_{0}}{V}\right)}^{1/3}-\left(f+1/2\right)\left(\frac{V_{0}}{V}\right)\right]+\frac{2\left[\ln\left(1-\phi \right)+\phi +\phi^{2}/2\right]}{\phi^{2}}$$

**Figure 2:**
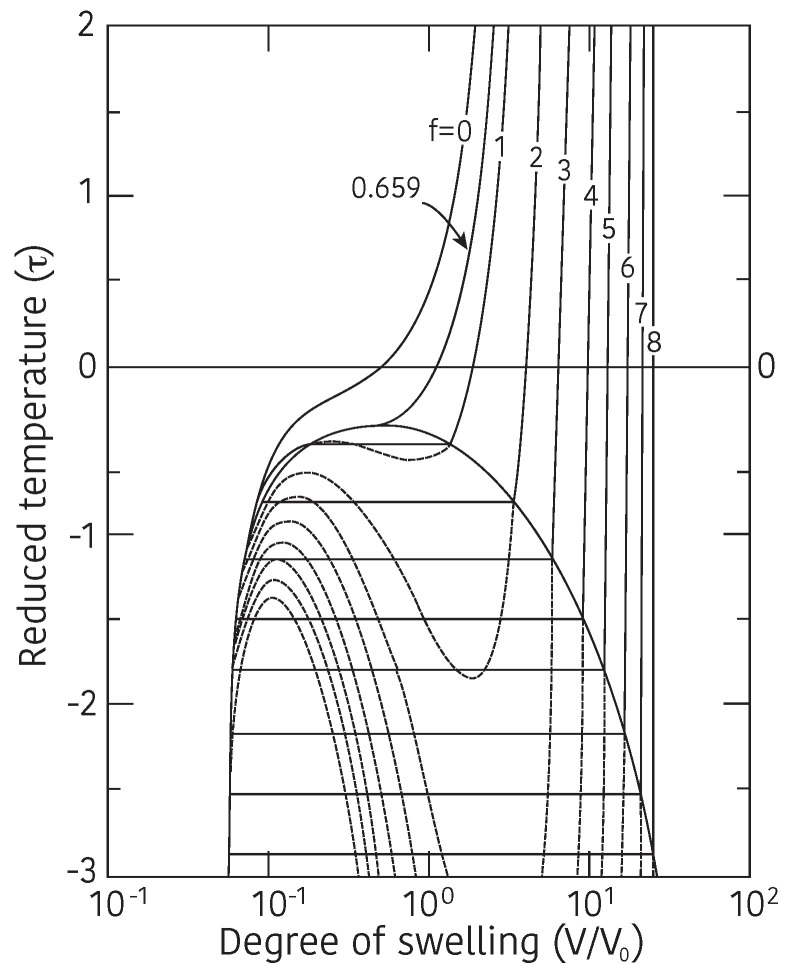
The theoretical swelling curve of gel calculated by Equation (8). The swelling curves are calculated for various values of ionic component on the active chain of the gel, *f*. Reproduced with permission from Ref. [8]. © 1986, The Physical Society of Japan.

The left hand member of this equation corresponds to the reduced temperature, τ, that depends only on the temperature, *T*, and the free energy of interaction between polymer and solvent, ΔF. It is clear from above Equation (8) that the change of temperature and the change of quality of the solvent causes the same result against the change of the volume of gel if proper solvent is chosen, namely, the variation in temperature and the variation in the quality of the solvent are equivalent. By using the Equation (8), the equilibrium swelling ratio of the gel, $V/V_{0}$, can be calculated as a function of the reduced temperature, τ=1−ΔF/kT, and results are given in Figure 2. The theoretical swelling curves of the gel, that given in Figure 2, correspond to the gels of various ionization, *f*. Some important results are deduced from the series of swelling curves of the gel shown in Figure 2. First of all, the swelling curve of the non-ionic gel, f=0, is a continuous function of the reduced temperature. Then, it becomes discontinuous as increasing the degree of ionization, *f*. It is enough to ionize one segment in the polymer chain, f=1, to induce the discontinuous volume change to the gel. It is also clear that the discrete volume change at the transition point becomes larger as increasing the degree of ionization of the polymer chain. Finally, the reduced temperature at the volume phase transition point becomes lower with the degree of ionization of the polymer chain. These results well explain the experimental results of the swelling behavior of the ionized poly(acrylamide) gel that shown in Figure 1.

#### 2.2.4. Critical Conditions for Discontinuous Volume Phase Transition

The logarithmic term in the theoretical swelling curve of gel, Equation (8), is expanded for further discussion.

(9) $$1-\frac{\Delta F}{kT}=\frac{2\nu \upsilon_{0}}{\phi^{2}}\left[{\left(\frac{\phi }{\phi_{0}}\right)}^{1/3}-\left(f+1/2\right)\left(\frac{\phi }{\phi_{0}}\right)\right]-\frac{2\phi }{3}$$

Scaling of above equation yields as

(10) $$\tilde{\tau }=S\left({\tilde{\rho }}^{-5/3}-\frac{{\tilde{\rho }}^{-1}}{2}\right)-\frac{\tilde{\rho }}{3}$$

where

(11) $$\tilde{\tau }=\frac{{\left(2f+1\right)}^{3/2}}{2\phi_{0}}\left(1-\frac{\Delta F}{kT}\right)$$

and

(12) $$\tilde{\rho }={\left(2f+1\right)}^{3/2}\left(\frac{\phi }{\phi_{0}}\right)$$

represent the scaled reduced temperature and scaled density of the polymer network, respectively.

The parameter, *S*, in Equation (10) is given as follows.

(13) $$S=\left(\frac{\nu \upsilon_{0}}{\phi^{3}}\right){\left(2f+1\right)}^{4}$$

In this approximation, the shape of the reduced equation of state is determined only by a single parameter, *S*. *S* solely determines whether the volume change is continuous or discontinuous. Theoretical calculation suggests a critical value of $S=S_{0}=243$ above which the phase transition is discontinuous and below which it is continuous.

The value of *S*, which determines the swelling curve of the gel uniquely, can be rewritten further by using the parameters that describe the segment of polymer chain. Let us consider an effective polymer chain consisting of *n* freely jointed segments of radius *a* and persistent length *b*. The volume of solvent is assumed to be $a^{3}$. When the interaction is neglected among the segments constituting the polymer, the average end-to-end distance of the chain is given as $R∼bn^{1/2}$. Since number of network chain in a unit volume of gel is ν∼1/R, then, the parameter *S* is written as follows.

(14) $$S={\left(\frac{b}{a}\right)}^{3}{\left(2f+1\right)}^{4}\;$$

This equation shows that the shape of the swelling curve of gel is uniquely determined by two physical factors. The one is b/a and the other is *f*. The value b/a represents the *stiffness* of the polymer chain and *f* is the number of the ionized group in the polymer chain. In summary, the volume change of the gel at the phase transition becomes larger and the transition temperature becomes lower when the polymer chain becomes stiff and/or the number of ionized group on the chain becomes lager. These two parameters are related through Equation (14).

#### 2.2.5. Volume Phase Transition in Various Gels

After the discovery of the volume phase transition in poly(acrylamide) gel, extensive experimental studies were made to confirm the theoretical description of the volume phase transition phenomena of gel. Tanaka classified the volume phase transition of gel into following four classes according to the driving forces of the phase transition.

1. Van der Waals interaction.
2. Hydrophobic interaction.
3. Hydrogen bond.
4. Electrical interaction between charges.

Here, we address some important experimental studies corresponding to the classifications briefly.

The phase transition behaviors of poly(acrylamide) gel are given in Figure 1 in detail. Tanaka classified the interaction that controlled the phase transition of poly(acrylamide) gel as van der Waals type because it plays important role in establishing the mean field theory of the volume phase transition of gel just like a van der Waals gas was.

**Figure 3:**
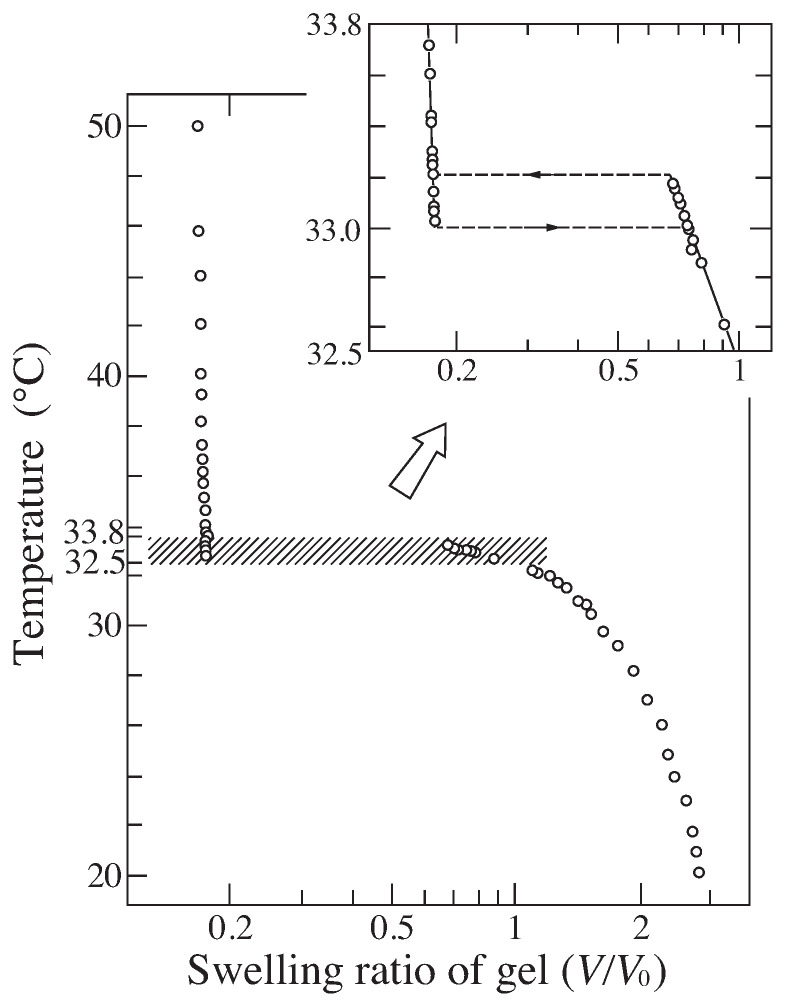
The swelling curves of non-ionic poly(N-isopropylacrylamide) gels. Reproduced with permission from Ref. [8]. © 1986, The Physical Society of Japan.

**Figure 4:**
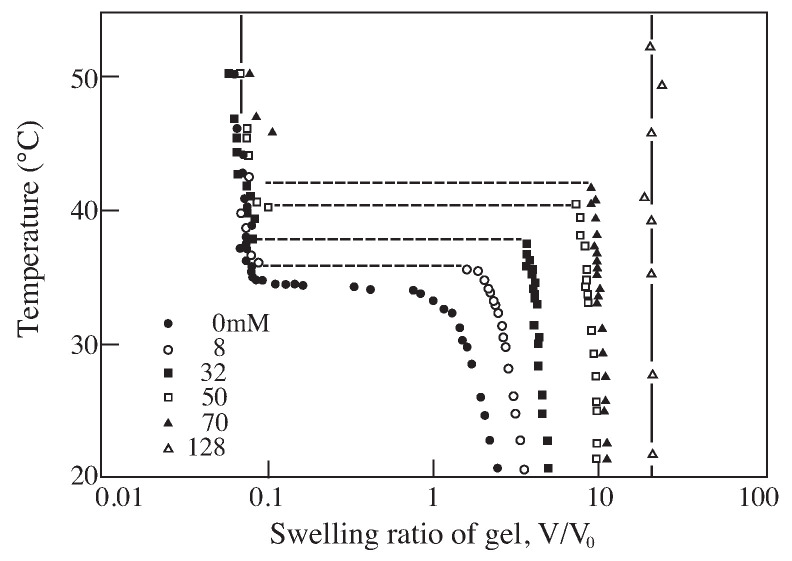
The swelling curves of ionic poly(N-isopropylacrylamide) gels. The gels are ionized by co-polymerization of a desired amount of sodium acrylate. Reproduced with permission from Ref. [8]. © 1986, The Physical Society of Japan.

**Figure 5:**
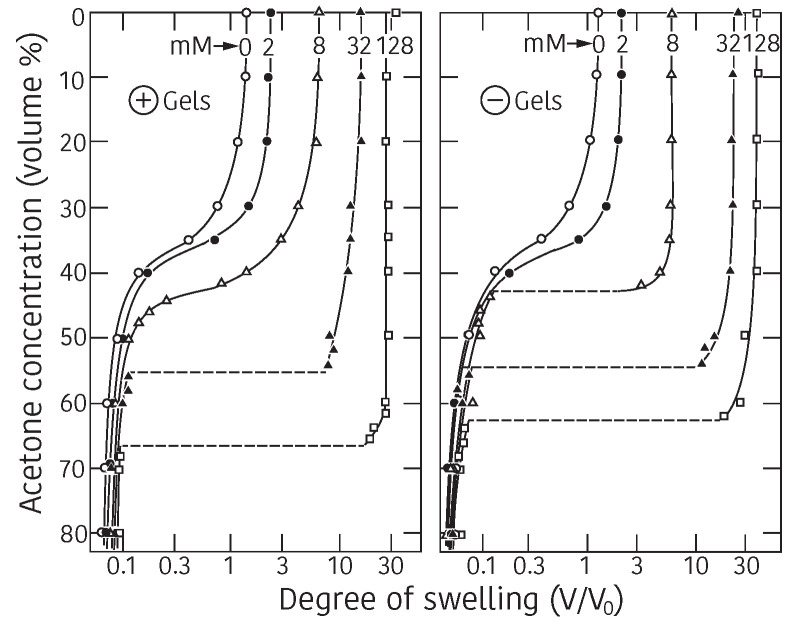
The swelling curves of positively charged gels and negatively charged gels. The desired amounts of (methacrylamidopropyl)trimethylammonium chloride is co-polymerized in the case of positively charged acrylamide gel. On the other hand, sodium acrylate is introduced into the polymer network of acrylamide by co-polymerization. Reproduced with permission from Ref. [8]. © 1986, The Physical Society of Japan.

According to the theory, Equation (14), the discontinuous volume phase transition of gel is observable whenever the polymer chain is stiff enough even though the gel is not ionized, f=0. A typical example of such system is poly(N-isopropylacrylamide) gel that is shown in Figure 3 [9]. It was found that this gel collapses at higher temperature and swells at lower temperature. The volume of poly(N-isopropylacrylamide) gel decreases discontinuously when the temperature is raised to 33.2 °C in pure water. The phase transition of poly(N-isopropylacrylamide) gel occurs due to the hydrophobic interactions between bulky side group of the polymer chain; N-isopropyl group. The fact that poly(N-isopropylacrylamide) gel shows the volume phase transition under pure water aids the further systematic studies of the volume phase transition of gel. For instance, the effects of the ionization on the volume phase transition of gel are clearly shown in the co-polymer gels of N-isopropylacrylamide and acrylic acid as shown in Figure 4 [10]. The characteristic behaviors of the swelling curves can be clearly observed in this figure. According to the theory, the phase transition behavior of the gel is independent of the sign of the charge. The expected results were obtained and shown in Figure 5 [11].

## 3. Dynamic Property of Gel

The polymer chain that constructs the polymer network of gel is flexible and it fluctuates in time and space even in the equilibrium state. The fluctuations of the network causes the fluctuations of the refractive index that scatters the light. It is shown both theoretically and experimentally that the time-correlation function of the fluctuation of scattered light intensity is expressed by the diffusion of the polymer network of the gel. Besides, the elasticity of the the polymer network and the diffusion coefficient of the polymer network are determined from the intensity of the scattered light and the decay rate of the fluctuation, respectively. On the other hand, the macroscopic swelling and shrinking processes of the gel are analyzed by the equation of motion of the gel. We will see that the results obtained from the light scattering experiments and that from the macroscopic swelling experiments yields the consistent picture for the dynamics of the gel.

### 3.1. Collective Diffusion of Polymer Network

Let us consider a unit cube of the polymer network of a gel alone with the density ρ. The polymer network of gel is regarded as a uniform elastic material. Then, the displacement of the unit cube from the average position r is expressed by the displacement vector u=u(r,t). The displacement vector is governed by the wave equation.

(15) $$\rho \frac{\partial^{2}u}{\partial t^{2}}=E_{l}\frac{\partial^{2}u}{\partial r^{2}}$$

Here, represents the longitudinal modulus of the network. The polymer network, however, $E_{l}$ moves in the sea of solvent in the gel. Therefore, the frictional force due to the solvent, which we assume to be proportional to the velocity of polymer chain ∂u/∂t, affects the motion of the chain. The wave Equation (15) above is, then, modified to be follows.

(16) $$\rho \frac{\partial^{2}u}{\partial t^{2}}=E_{l}\frac{\partial^{2}u}{\partial r^{2}}-f\frac{\partial u}{\partial t}$$

The inertia term in Equation (16) is much smaller than the two terms in the right hand member in usual case of gels. Neglecting the inertia term, we obtain following diffusion equation.

(17) $$\frac{\partial u}{\partial t}=D_{\mathrm{coop}}\frac{\partial^{2}u}{\partial r^{2}}$$

The Equation (17) is the collective diffusion equation of gel and $D_{\mathrm{coop}}=E_{l}/f$ the collective diffusion coefficient of gel. Equation (17) indicates that the polymer chain of the network collectively diffuses with a diffusion coefficient $D_{\mathrm{coop}}$.

### 3.2. Swelling Behavior

The collective diffusion equation of the gel, Equation (17), was solved under the initial and the boundary conditions for a spherical gel and compared with the experimental results of the kinetics of the swelling in spherical gels [12]. It is found that the collective diffusion coefficient of gel becomes the order of $D_{\mathrm{coop}}∼10^{-7}$ cm${}^{2}$/s. The results indicate that the collective diffusion coefficient of the polymer network is about 1/100 of the smaller molecules such as the monomer, which is the order of $D∼10^{-5}$ cm${}^{2}$/s. This is a direct experimental demonstration of the collective motion of the polymer network. It is clear from the dimension analysis of Equation (17) that the characteristic time that governs the swelling process, [Time], is proportional to the square of the characteristic length scale, *L*.

(18) $$\left[\mathrm{Time}\right]\propto \frac{L^{2}}{D_{\mathrm{coop}}}$$

**Figure 6:**
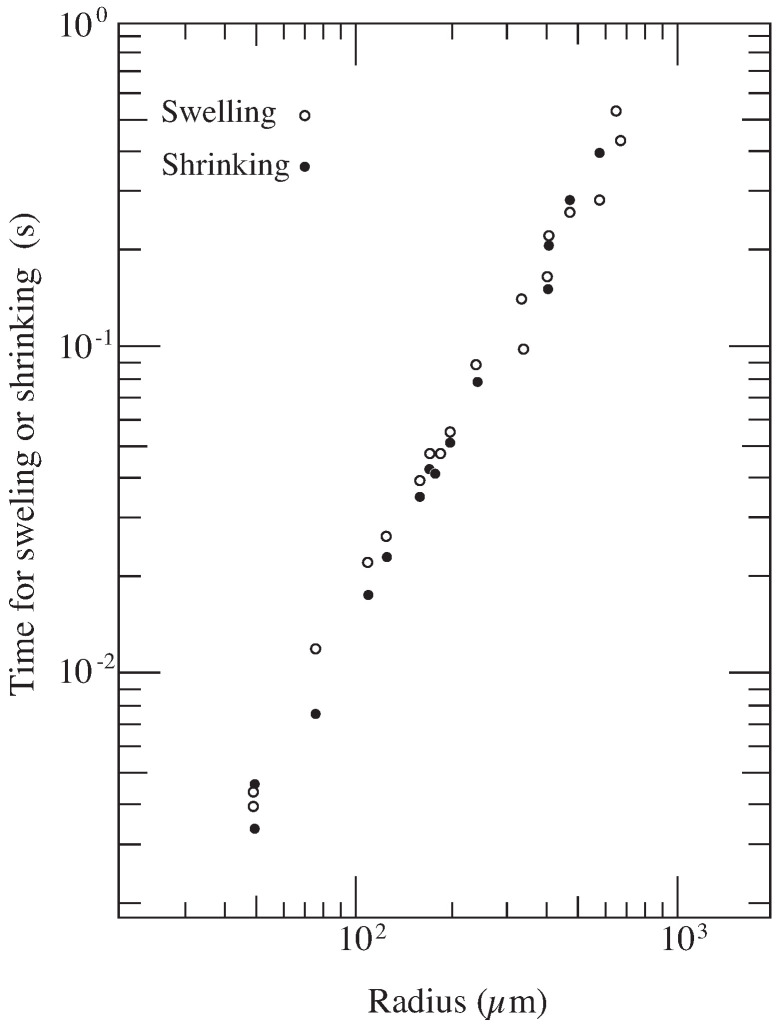
Swelling time and shrinking time of gels. The measurements are made in poly(N-isopropylacrylamide) gel particles [13]. Reproduced with permission from Ref. [8]. © 1986, The Physical Society of Japan.

The results indicate that the response time of the swelling and collapse against stimuli becomes smaller if the size of the gel becomes smaller as shown in Figure 6. For instance, the swelling time of the gel about 1cm size is of the order of one day while the swelling time of the gel of 1 μm size becomes of the order of 10${}^{-3}$ s. The results are in good agreement with the Equation (18) [13]. The result obtained from these swelling experiments of the gel provide the important information not only for designing the experimental study of the gel but also for the practical use of the gel in industry.

### 3.3. Light Scattering from Collective Mode of Gel

**Figure 7:**
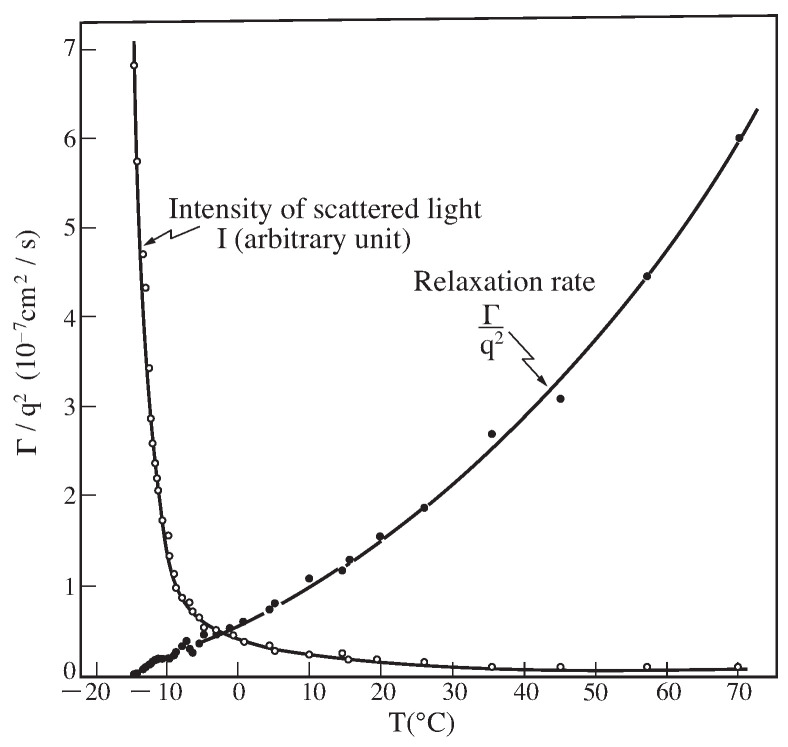
The critical slowing down in poly (acrylamide) gel observed by the light scattering measurements. Reproduced with permission from Ref. [8]. © (1986) The Physical Society of Japan.

The collective diffusion mode of the gel gives rise the light scattering. The space–time correlation function of the scattered light electric field is proportional to the spatial Fourier transform of the time-correlation function of the density fluctuations. It is given by an exponential decay function with the amplitude that is inversely proportional to the longitudinal modulus of the gel, $E_{l}=K+\frac{4\mu }{3}$. Here, *K* and μ are the bulk modulus and the shear modulus of the polymer network, respectively. On the other hand, the decay rate is proportional to the collective diffusion coefficient.

(19) $$<E\left(q,t\right)E^{*}\left(q,0\right)>∼\frac{kT}{E_{l}}\exp\left(-\Gamma t\right)$$

(20) $$D_{\mathrm{coop}}=\frac{\Gamma }{q^{2}}=\frac{\left(K+\frac{4}{3}\mu \right)}{f}$$

where *f* represents the friction coefficient between the polymer network of the gel and the solvent. Here, q represents the scattering vector. The result, above Equation (19), was confirmed by measuring the correlation function of light scattered from poly(acrylamide) gel in water [14]. The temperature dependence of the light scattering revealed the critical behavior in poly(acrylamide) gel as shown in Figure 7 [15]. Tanaka made this study before the discovery of the volume phase transition of gel. The intensity of the scattered light diverges and the relaxation time slows down to zero as the temperature approaches the critical point. The results indicate that the fluctuations of polymer network increases to infinity and the relaxation rate slows down infinitely. The gel becomes opaque as a results of non-uniform spatial density distribution of the polymer network in the vicinity of the critical point. The opacity of the gel, however, disappears and the gel becomes transparent reversibly when the distance from the critical point is increased. It is found that the divergence of the fluctuation of the polymer network is well explained by the mode-mode coupling theory [16].

### 3.4. Critical Phenomena

It is well known that the critical phenomena can be seen in a wide variety of material systems expanding from gas to solid. In most case of gel, we recognize it by the appearance of the strong opalescence. The gel, thus, gets opaque near the critical point. The light scattering results indicate that the relaxation rate decreases toward zero in the vicinity of the critical point as shown in Figure 7. Thus, we expect

(21) $$\lim_{T\to T_{c}}D_{\mathrm{coop}}\to 0.$$

The longitudinal modulus of the gel, which is independently determined from the intensity of the scattered light, also becomes zero near the critical point, Equation (20).

(22) $$\lim_{T\to T_{c}}E_{l}\to 0$$

**Figure 8:**
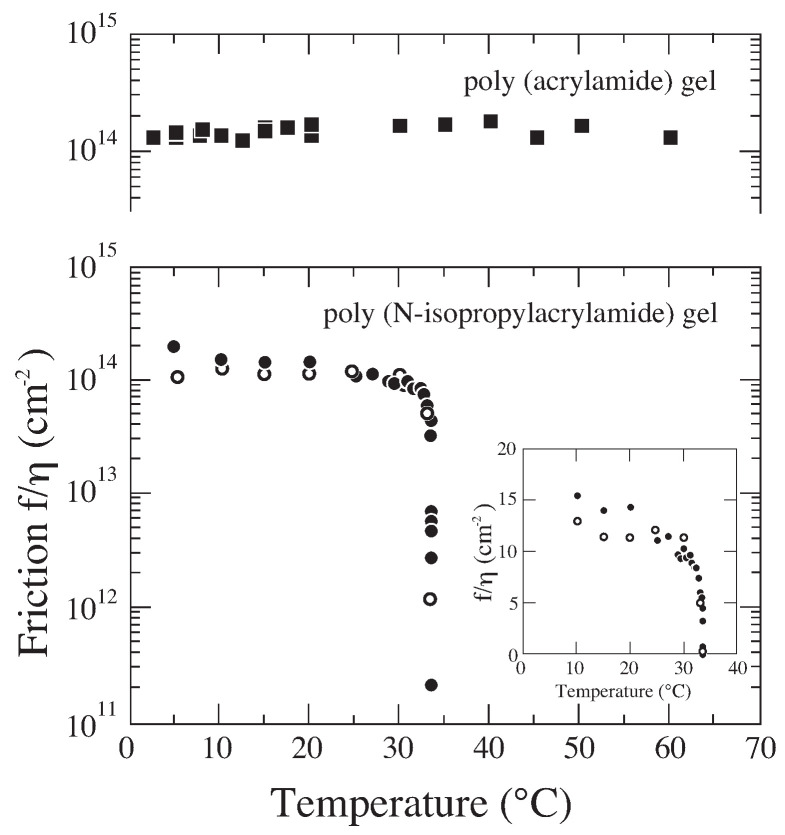
Reversible decrease of gel-solvent friction observed by the mechanical measurements. The inset shows the linear plot of the friction.

These results, Equations (21) and (22), are consistent because $D_{\mathrm{coop}}=E_{l}/f$. It is, however, extremely desirable to measure the friction coefficient of gel in the vicinity of the critical point of the gel, though it can be estimated from the collective diffusion coefficient and the longitudinal elastic modulus of the gel by Equation (20). The experimental study of the critical behavior of the friction coefficient of the gel was made much later than the finding of the critical phenomena of the gel. A new apparatus for the friction measurement should be constructed, and then, the frictional properties of gels were studied [17,18]. The results are given in Figure 8. In this figure, the friction coefficient of the gel is normalized by the viscosity of water, f/η, because the flow rate of water depends on the viscosity of flowing fluid. The friction of the poly (acrylamide) gel is almost constant in the temperature region studied. In contrast, the friction of poly(N-isopropylacrylamide) gel decreases about three orders of magnitude in the vicinity of the volume phase transition temperature of this gel, T∼33 °C. We finally find the critical behavior of friction

(23) $$\lim_{T\to T_{c}}f\to 0.$$

The friction of the gel, therefore, decreases to zero in the vicinity of the volume phase transition point of the gel. The fluid of the gel easily flows through the gel when the critical point is approached. Here, we obtained the complete set of the critical behaviors of the gel. Namely, the longitudinal modulus of the gel becomes smaller in the vicinity of the critical point, $E_{l}\to 0$. The gel gets opaque because the intensity of the scattered light diverges as $I\propto E_{l}^{-1}$. At the same time, the fluctuations of the density of the polymer network becomes slower, $D_{\mathrm{coop}}\to 0$. The density fluctuation of the polymer network creates both the dense regions and the dilute regions of polymer network within the gel. Since the collective diffusion coefficient of the gel becomes zero in the vicinity of the critical point, the distribution of the dense regions and the dilute regions becomes spatially pinned. The solvent of the gel, therefore, flows through the gel easily because the dilute regions serve as the open pore for the solvent flow, f→0. This is a rough picture of the critical phenomena in the gel.

**Figure 9:**
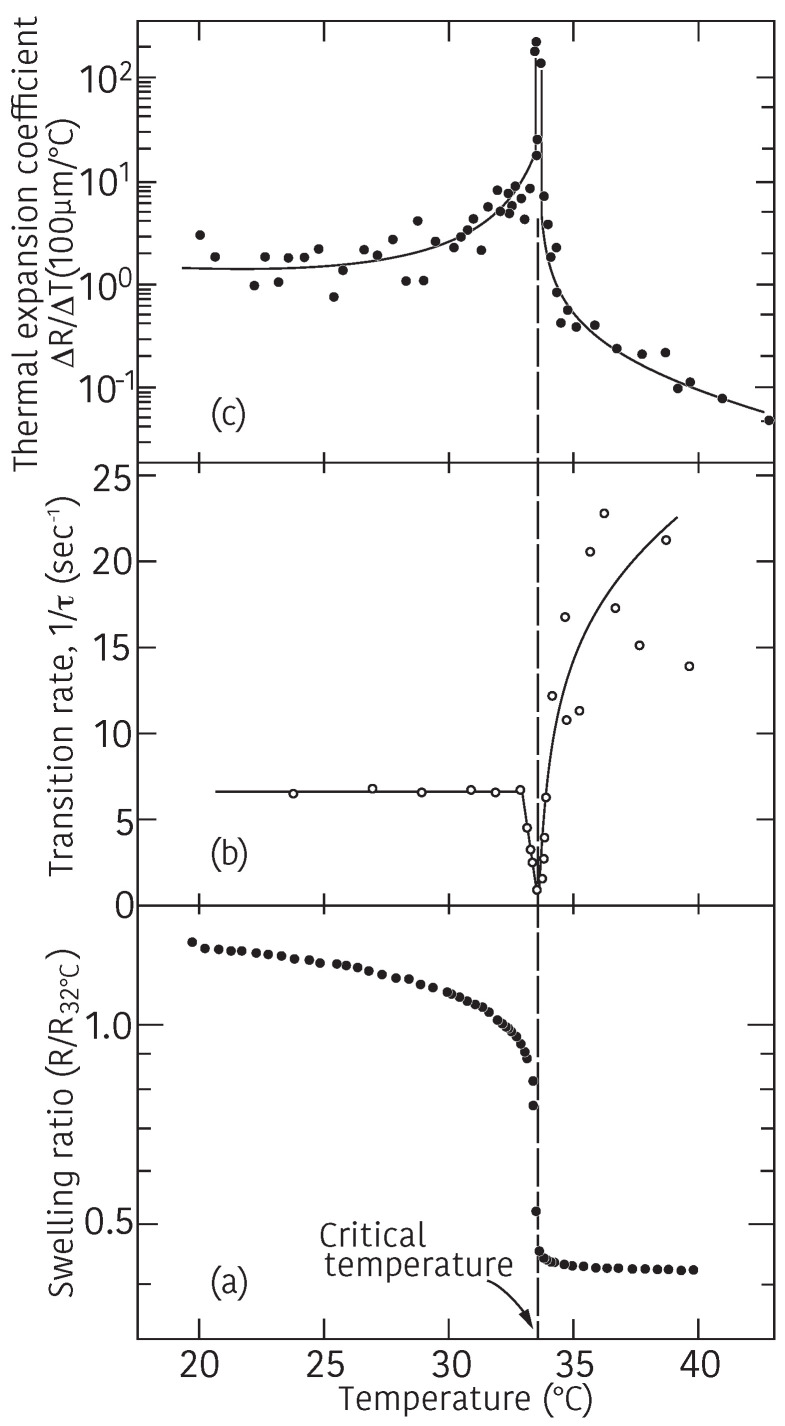
Critical kinetics of the gel. The swelling curve of the gel (**a**); the temperature dependence of the transition rate of the volume change (**b**); and the temperature dependence of the thermal expansion coefficient of the gel (**c**). Reproduced with permission from Ref. [8]. © 1986, The Physical Society of Japan.

Finally, we present the experimental results on the critical kinetics of swelling and shrinking of the gel. The light scattering from the gel indicates that the collective diffusion coefficient of the gel becomes zero when the gel approaches the critical point, Equation (21). It may be natural to ask how the swelling and shrinking of the gel are affected near the critical point of the gel. The experiments are made on the spherical poly(N-isoropylacrylamide) gel of sub-millimeter in size [13]. The results are given in Figure 9. It is clear from the results that the transition rate strongly depends on the temperature. Thus, the total rate of the volume change depends both on the initial position and final position of the gel in the phase diagram. The swelling and the shrinking of the gel become infinitely slow at the critical point where the volume of the gel shows the discontinuous transition.

## 4. Concluding Remarks

We quickly reviewed the research work of Professor Toyoichi Tanaka here. We mainly focused our attention to the early works of the volume phase transition of the gel. Although the theory that he had constructed is of mean field type, it explains the experimental results rather well. Therefore, we believe that it will be still a good guideline for young scientist who entering into the gel science. Because of the limited space, many exotic results were not addressed here. We hope readers to cite other works published by Tanaka, which could not cited here, for deepen their knowledge about the science of gels.

In the end of this review, we would like to address about some future works of gels. First one is related to the critical phenomena. In the section of critical phenomena, we found that physical parameters tend to disappear at the critical point, namely,

$$\begin{array}{cc} \; & \lim_{T\to T_{c}}D_{\mathrm{coop}}\to 0,\\ \; & \lim_{T\to T_{c}}E_{l}\to 0,\\ \; & \lim_{T\to T_{c}}f\to 0. \end{array}$$

The results intuitively depicts the state of the gel at the critical point very well. The results are, however, still qualitative because these results were obtained in different gels of acrylamide and N-isopropylacrylamide. Therefore, systematic measurements of $D_{\mathrm{coop}}$, $E_{l}$, and *f* in the same gel are required for further quantitative understanding of the critical phenomena of the gel. It may be possible to discuss the relationship between these parameters theoretically through the critical exponents for these parameters [19].

Second subject is related to the pattern formation in the gel. It has been reported that beautiful patterns are formed both in swelling and shrinking processes of the gel. The swelling pattern of the gel were analyzed and results suggest that the mechanical instability at the surface of the swelling gel plays important roles for the formation of the swelling pattern [20]. In contrast, the shrinking patterns of the gel are yet to be analyzed in detail. The gel forms various patterns in the shrinking process. It is only suggested that the relationship between the final patterns and the shrinking conditions in a form of the “*phase diagram of shrinking patterns*” [21]. Few experimental studies were reported in which the confocal laser scanning microscopy is employed. Such studies suggest that the destruction of the polymer network occurs during the formation of the shrinking patterns [22]. Besides, in the case of the bubble formation process, the observation results strongly suggest that the constant volume conditions in the initial state of the shrinking process plays essential roles for the pattern formation of the gel. It further suggest that the shrinking pattern formation process may be related to the non-equilibrium steady state of the shrinking gel [23].

**Figure 10:**
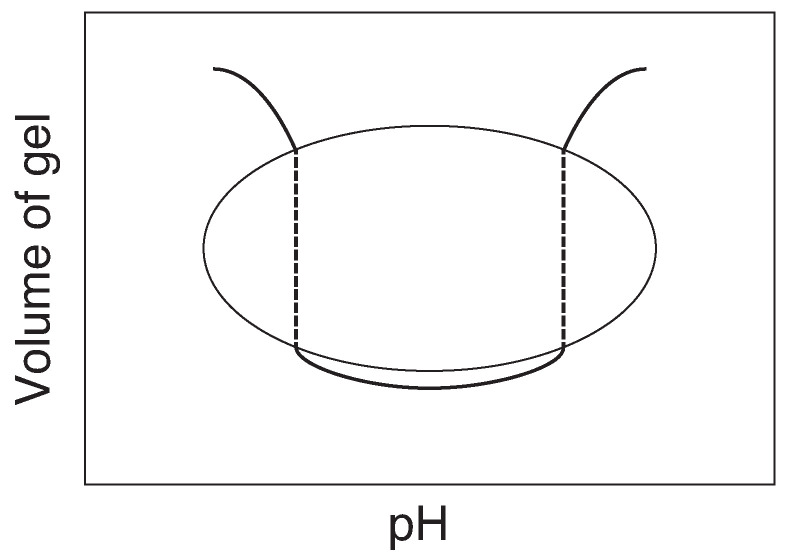
A simple estimation of the swelling curve of gel that contains both the positive charges and the negative charges.

The third example is related to the last project of Tanaka. When we discuss in the section of the volume phase transition of various gels, we left the experimental results about the volume phase transition of gel due to the electrical charges. It may be natural to design the gel that contains both positively charged segments and negatively charged segments to clarify the effects of the interaction between electrical charges of polymer chain on the volume phase transition of the gel. The concentration of proton, i.e., pH, may also be the natural choice of the external variable to observe the volume phase transition in such gels. It is reasonably assumed that the gel that contains both the positively charged segments and the negatively charged segments swell both at lower and higher pH regions and it collapses into compact state in the intermediate pH region because we know that either positively and negatively charged gels swell lower pH region and higher pH region, as shown in Figure 10 schematically. He, however, discovered entirely new volume phase transition phenomena in the gels that contains both the positively charged segments and the negatively charged segments, namely, *multiple volume phase transition of the gel* [24]. The gel shows many stable swollen state against pH change. The phenomenon is believed to occur by the cooperative interaction between the hydrogen bonding, the repulsive force between the same charges, and the attractive force between opposite charges. The similar behaviors were observed in the chemically cross-linked biopolymer gels. A totally new idea was born in Tanaka’s mind through the studies of the multiple phases of gel. He found the similarity of the origin between the multiple volume phase transition of gel and the structure transition in the heteropolymer system. He, then, moved to study the phase transition of the heteropolymer systems theoretically [25,26]. After these studies, his idea was expanded to establish the molecular recognition system by the heteropolymer gel with the idea of imprinting; such system are known only in biological molecules as proteins. He wanted to prepare a heteropolymer gel in which some information is imprinted within its structure in such a way that some degree of molecular self-assembly would be achieved in the shrunken state of the gel. He actually make significant progress in this direction [27,28]. It was demonstrated experimentally in some heteropolymer gels that imprinted molecular information leads to minimize the frustrations in the gel. Nevertheless, this program remains incomplete. Some researchers pointed out that the imprinting of information has very little chance to succeed and some were skeptical about his idea. However, I believe that Tanaka was seeing the answer for the problem of “*what is life*” behind the research of heteropolymer gels. Tanaka sometime expressed this problem as “*the origin of life*”. Apart from such a big problem, we believe that above ambitious studies will open a new insight into the gel science as well as the life science. Although the path to success may be narrow and steep, young scientists should try it. The big scientific achievements are not found on convenient paved roads as suggested by Feynman in the title of his book, “Perfectly reasonable deviations from the beaten track”. Tanaka left many seeds of science to be solved. I believe that solving of these problems will contribute to the deeper understanding of the gel science.
